# Are high residual chlorhexidine skin concentrations associated with improved clinical outcomes? Lessons from the CLEAR trial

**DOI:** 10.1017/ice.2026.10424

**Published:** 2026-05

**Authors:** Roya Khoja, Tabitha D. Catuna, Gabrielle M. Gussin, Kevin Nguyen, Raveena D. Singh, Mary K. Hayden, Loren G. Miller, Susan S. Huang

**Affiliations:** 1 Division of Infectious Diseases, University of California Irvine School of Medicine, Irvine, USA; 2 Division of Infectious Diseases, Rush University Medical Center, Chicago, USA; 3 Division of Infectious Diseases, Lundquist Institute for Biomedical Innovation at Harbor– UCLA Medical Center, USA; 4 David Geffen School of Medicine at the University of California, Los Angeles, CA, USA

## Abstract

Residual chlorhexidine gluconate (CHG) skin concentrations are thought to improve disease prevention. In the Changing Lives by Eradicating Antibiotic Resistance (CLEAR) trial, posthospitalization decolonization of MRSA carriers reduced MRSA infection and all-cause infection, but higher residual CHG concentrations did not improve outcomes. CHG concentration may indicate bathing quality, but high residual concentrations may not be necessary for benefit.

## Introduction

Chlorhexidine gluconate (CHG) is a topical antiseptic that reduces skin microbial bioburden, bloodstream infections, and multidrug-resistant organisms (MDROs) in large-scale trials of hospitalized patients, postdischarge patients, and nursing home residents.^1–3^

CHG binds to skin proteins for a 24-hour germicidal effect.^1,4^ A 4% rinse-off shower or 2% leave-on bed bath imparts 40,000 mcg/mL and 20,000 mcg/mL of CHG to the skin, respectively, far exceeding the minimum inhibitory concentration (MIC) of Gram-positive (e.g., *Staphylococcus aureus* MIC_90_ = 5 mcg/mL)^5–6^ and Gram-negative pathogens (∼150 mcg/mL).^7^ Nevertheless, some believe CHG skin concentrations must persistently exceed these MICs for 24 hours postbathing for clinical benefit. We tested this hypothesis within a randomized-controlled trial of serial decolonization to reduce methicillin-resistant *Staphylococcus aureus* (MRSA) and all-cause infection in postdischarge MRSA carriers.^2^

## Methods

We conducted a secondary analysis of the Changing Lives by Eradicating Antibiotic Resistance (CLEAR) trial,^2^ to evaluate whether higher residual CHG skin concentrations were associated with improved clinical outcomes. The CLEAR intervention involved postdischarge 4% rinse-off CHG antiseptic soap for showering, 0.12% CHG mouthwash, and 2% nasal mupirocin given Monday-Friday twice-a-month for 6 months to discharged MRSA carriers. Participants were followed for one-year outcomes of MRSA infection and all-cause infection. In addition, MRSA carriage was assessed by body site sampling at enrollment and 1, 3, 5, and 9 months postdischarge.

Participants who showered with CHG within 24 hours of their visit underwent CHG skin concentration sampling. A 10 x 10 cm^2^ area on the deltoid and abdomen were sampled with cotton-tipped swabs (Bio-Swab, Arrowhead Forensics, Lenexa, KS). CHG was detected using a quantitative colorimetric assay based on serial two-fold dilutions and reported as 0, <4.8 (given value of 2.4 for analysis), 4.8, 9.8, 19.5, 39.1, 78.1, 156.3, 312.5, and 625 mcg/ml.^6^ Of the two sites, the higher CHG concentration was analyzed.

Residual CHG concentrations were plotted by participant sex, age, MRSA carriage at the visit, and trial outcomes of MRSA infection and all-cause infection occurring postvisit. Bivariable and multivariable analyses were performed using logistic regression (SAS9.4, Cary, NC). This study was approved by the University of California Irvine Institutional Review Board.

## Results

We obtained CHG samples from 137 participants in the decolonization arm, representing follow-up visits in month 1 (*N* = 75, 54.7%), 3 (*N* = 45, 32.8%), and 5 (*N* = 17, 12.4%). Half were female (*N* = 66, 48%) and median age (range) was 56 (22–89) years. Age was categorized into 3 tiers: <50 (*N* = 40 (29%), 50–64 (*N* = 53, 39%), and 65+ years (*N* = 44, 32%).

**Figure 1.**
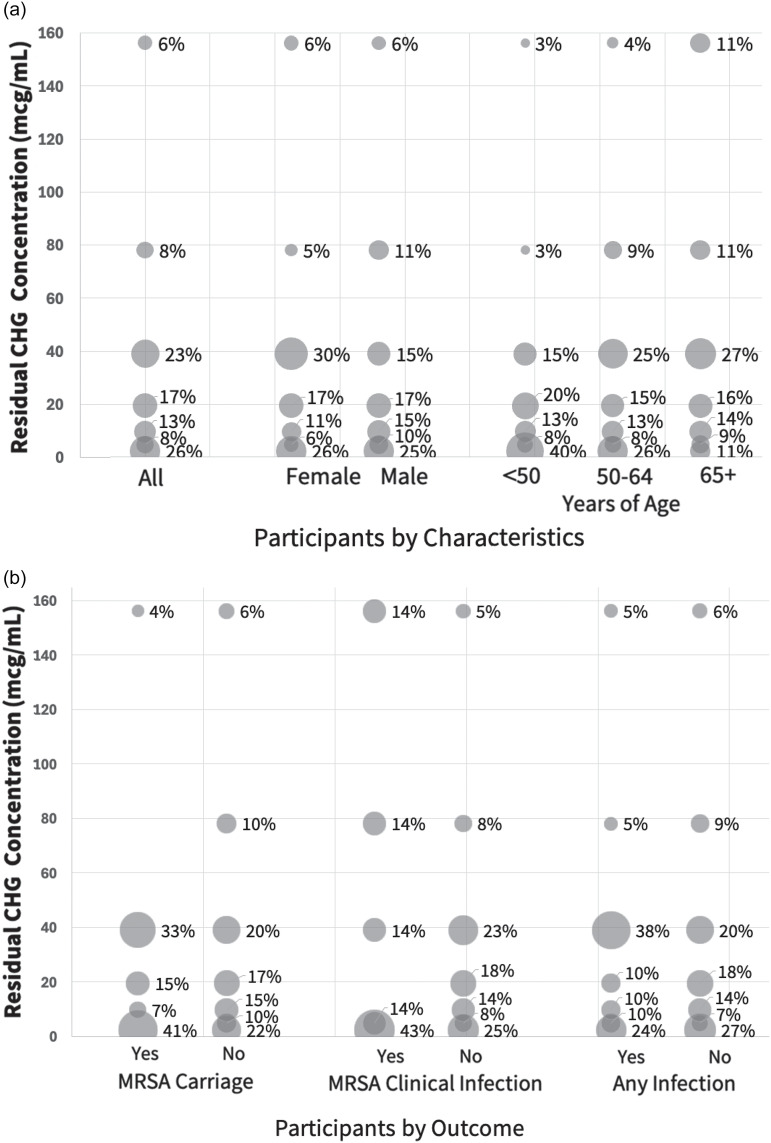
Residual CHG skin concentration by participant characteristics and outcomes. Plotted relationship between postshower residual chlorhexidine gluconate (CHG) skin concentration and select participant characteristics or trial outcomes. Bubble size is proportional to the number of participants within each category. Panel 1a shows the relationship between CHG concentration and sex and age. Median (range) residual CHG skin concentration among females was 19.5 mcg/mL (0–625), and 9.8 mcg/mL (0–312.5) among males. Median concentrations for those <50 years were 9.8 mcg/mL (0–156.3); 50–64 years, 19.5 mcg/mL (0–312.5); and 65+ years, 29.3 mcg/mL (0–625). Panel 1b shows the relationship between CHG concentration and outcomes of carriage or infection. Median (range) CHG concentrations were 19.5 mcg/mL (0–312.5) among MRSA carriers and 19.5 mcg/mL (0–625) among non-carriers; 4.8 mcg/mL (0–312.5) among those who developed MRSA infection and 19.5 mcg/mL (4.8–625) among those who did not; and 19.5 mcg/mL (0–312.5) among those who developed any infection and 19.5 mcg/mL (0–625) among those who did not.

The median residual CHG concentration across all participants was 19.5 mcg/mL (range 0–625), without variation by visit month (Supplement). Figure 1a displays residual CHG concentration by demographics. Median concentrations were 19.5 mcg/mL (0–625) among females and 9.8 mcg/mL (0–312.5) among males; for those <50 years, 9.8 mcg/mL (0–156.3); 50–64 years, 19.5 mcg/mL (0–312.5); and 65+ years, 29.3 mcg/mL (0–625). Only age was significantly associated with higher residual CHG concentration (OR = 2.62, *P* = .02) on bivariable modeling.

When displaying residual CHG concentration by clinical outcomes (Figure 1b), 27 (20%) were MRSA carriers at the CHG skin swab visit, 7 (5%) experienced subsequent MRSA infection, and 21 (15%) experienced subsequent all-cause infection. In bivariable and multivariable modeling, neither participant characteristics nor higher residual CHG concentrations (high: ≥19.5 mcg/mL versus low: <19.5 mcg/mL) were associated with MRSA carriage, MRSA infection, or all-cause infection (Table 1).

**Table 1.** A two-panel graphical display of CHG skin concentration by age and sex and by clinical outcomes of MRSA carriage and infections

Characteristic	MRSA carriage	MRSA infection	All-cause infection
Female	1.17 (0.49, 2.82)	0.82 (0.17, 3.97)	1.17 (0.46, 3.03)
Age (years)			
≤ 49	1.0	1.0	1.0
50–<65	0.25 (0.08, 0.78)	4.28 (0.46, 39.48)	0.94 (0.31, 2.85)
65+	0.70 (0.25, 1.93)	1.00 (0.06, 17.56)	0.57 (0.16, 2.05)
CHG^1^ ≥19.5 mcg/ml	0.97 (0.39, 2.40)	0.65 (0.13, 3.16)	1.30 (0.49, 3.43)

^1^CHG, chlorhexidine gluconate.

## Discussion

The benefit of CHG in reducing healthcare-associated infections, including bloodstream infections and MDROs, has been attributed to its antiseptic cleansing properties and residual germicidal effects lasting up to 24 hours.^5^ It is long believed that residual CHG skin concentrations are important to clear MDRO carriage and reduce infection in presurgical and hospitalized patients.^5–7^ In this secondary analysis of the CLEAR trial, we did not find a significant association between residual CHG skin concentrations and either MRSA carriage or infection. Nevertheless, the CLEAR trial demonstrated that decolonization with CHG resulted in a 30% reduction in MRSA infection and a 17% reduction in all-cause infection compared to controls (standard bathing).^3^ Thus, next-day residual skin concentration may not be important in this postdischarge population.

The lack of association between CHG skin concentration and clinical outcomes is notable because many participants had residual concentrations well below and well above the 5 mcg/ml MIC_90_ of the target pathogen (MRSA).^5–6^ Our findings challenge current assumptions that CHG concentrations must be persistently above the MIC to reduce infection risk from human pathogens. Clinical benefit may be predominantly due to direct application of CHG during a shower or bed bath. Participants in the CLEAR trial applied 4% CHG as shower soap, the equivalent of 40,000 mcg/mL to the skin. Because CHG is rapidly bactericidal and this concentration far exceeds the MICs of MDRO and non-MDRO healthcare-associated pathogens, reductions in MDRO carriage and infection may lie in the quality and comprehensiveness of direct application when bathing or showering. Moreover, the reduction in MDRO carriage at 24 hours postbath may be due to both direct germicidal activity and the time it takes for re-exposure and re-colonization to occur rather than a 24-hour persistent germicidal effect. Another possibility is that detectable CHG on the skin underestimates its microbial benefit. Finally, CHG levels that minimize infection may differ in postdischarge patients versus hospitalized patients due to different behaviors, activities, and physical contacts.

Regardless of whether residual CHG concentration is of consequence, feedback of postbathing skin concentrations improves bathing quality, which varies across healthcare providers.^4–8^ Even CHG bathing by ICU nurses fails to achieve the known reduction in bloodstream infections without proper bathing training.^9^ Thus, it is notable that CHG bathing education provided to CLEAR participants achieved a 30% reduction in infection despite showering on their own at home. Participants were told to comprehensively soap the body twice using firm massage for 2 minutes outside the water stream before rinsing.

Nevertheless, high residual CHG skin concentrations may further enhance clinical benefit in a way not detectable with the population and outcomes of the CLEAR trial, or the sample size of this study. For example, the CHG MICs for Gram-negative bacteria and especially *Candidozyma auris* are higher than *S. aureus*.^7,10^ Additional limitations include the fact that CHG concentration was based on a convenience sample of those performing CHG bathing in the 24 hours prior to a follow-up visit. This may have favored those who showered more often on top of clinical trial volunteerism that would enhance adherence. Still, these bathing enhancements should have only improved our ability to detect a difference associated with residual CHG skin concentration. Another limitation is that concentrations were only assessed once, which may not have captured typical bathing behavior and quality. Finally, we did not link the CHG concentration to the MIC of the MRSA strain harbored by each participant. Nevertheless, representative sampling in the CLEAR trial found that nearly all MRSA strains (99.9%) had MICs under 8 mcg/mL.^2^

In summary, we did not find an association between postshower residual CHG skin concentrations and reductions in MRSA carriage or infection in the CLEAR trial. This suggests that CHG benefit may be predominantly imparted at the time of the bath or shower, with residual germicidal effects persisting despite what is detected on the skin, or attributed to the time it takes re-colonization to occur. Using residual CHG skin concentrations for training and ensuring bathing quality may be more important than the exact detected value for achieving the known clinical benefits of CHG.

## Supporting information

Khoja et al. supplementary material Khoja et al. supplementary material

## Supplementary material

The supplementary material for this article can be found at https://doi.org/10.1017/ice.2026.10424.

## References

[1] Huang SS. Chlorhexidine-based decolonization to reduce healthcare-associated infections and multidrug-resistant organisms (MDROs): who, what, where, when, and why? J Hosp Infect 2019;103: 235–243. doi:10.1016/j.jhin.2019.08.025. 31494130

[2] Huang SS , Singh R , McKinnell JA , et al. Decolonization to reduce postdischarge infection risk among MRSA carriers. N Engl J Med 2019;380:638–650. doi:10.1056/NEJMoa1716771. 30763195 PMC6475519

[3] Miller LG , McKinnell JA , Singh RD , et al. Decolonization in nursing homes to prevent infection and hospitalization. N Engl J Med 2023; 389:1766–1777. doi: 10.1056/NEJMoa2215254. 37815935 PMC13016440

[4] Popovich KJ , Lyles R , Hayes R , et al. Relationship between chlorhexidine gluconate skin concentration and microbial density on the skin of critically ill patients bathed daily with chlorhexidine gluconate. Infect Control Hosp Epidemiol 2012;33:889–896. doi: 10.1086/667371. 22869262 PMC3632447

[5] Edmiston CE , Krepel CJ , Spencer MP , et al. Preadmission application of 2% chlorhexidine gluconate (CHG): enhancing patient compliance while maximizing skin surface concentrations. Infect Control Hosp Epidemiol 2016;37:254–259. doi: 10.1017/ice.2015.303. 26708510

[6] Rhee Y , Hayden MK , Schoeny M , et al. Impact of measurement and feedback on chlorhexidine gluconate bathing among intensive care unit patients: a multicenter study. Infect Control Hosp Epidemiol 2023;44:1375–1380. doi: 10.1017/ice.2023.177. 37700540 PMC10859163

[7] Lin MY , Lolans K , Blom DW , et al. The effectiveness of routine daily chlorhexidine gluconate bathing in reducing Klebsiella pneumoniae carbapenemase-producing Enterobacteriaceae skin burden among long-term acute care hospital patients. Infect Control Hosp Epidemiol 2014;35:440–442. doi: 10.1086/675613. 24602954 PMC8381087

[8] Supple L , Kumaraswami M , Kundrapu S , et al. Chlorhexidine only works if applied correctly: use of a simple colorimetric assay to provide monitoring and feedback on effectiveness of chlorhexidine application. Infect Control Hosp Epidemiol 2015;36:1095–1097.doi: 10.1017/ice.2015.124. 26074153

[9] Noto MJ , Domenico HJ , Byrne DW , et al. Chlorhexidine bathing and health care–associated infections: a randomized clinical trial. JAMA 2015;313:369–378. doi:10.1001/jama.2014.18400. 25602496 PMC4383133

[10] Gussin GM , Singh RD , Shimabukuro J , et al. Candida auris and MRSA shedding during caregiving versus rest in nursing homes (Abstract 300),Society for Healthcare Epidemiology of America (SHEA) Spring Conference 2025. 2025.

