# Supplementary material for: Are high residual chlorhexidine skin concentrations associated with improved clinical outcomes? Lessons from the CLEAR trial
Source: Infect Control Hosp Epidemiol. 2026 Mar 26;47(5):522–5. doi: 10.1017/ice.2026.10424 (PMC13040246; doi:10.1017/ice.2026.10424)
Supplement: Khoja et al. supplementary material [file S0899823X26104243sup001.pdf]

**Supplement**

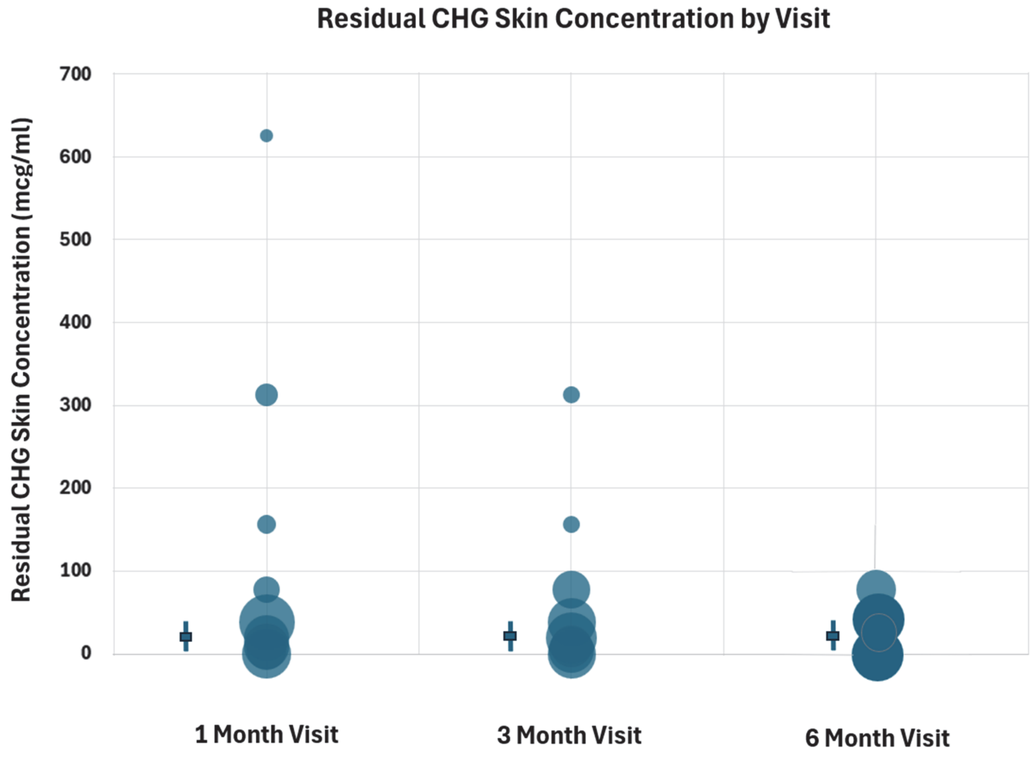


**Figure S1:** Graphic displaying residual CHG skin concentration by the follow up visit during which it was taken. Size of circles are proportional to the number of participants with that value. Median values (box) and their inter-quartile range (line) are shown next to the distribution of values at each visit. Medians and interquartile ranges were identical across all visits (median: 19.53 ug/ml (4.88, 39.06)).
